# Virtual Reality for the Management of Postoperative Pain and Anxiety in Children and Adolescents Undergoing Nuss Repair of Pectus Excavatum: Randomized Controlled Trial

**DOI:** 10.2196/80902

**Published:** 2026-03-10

**Authors:** Charlotte M Walter, Dillon Froass, Nora Bell, Lauren Haack, Chloe Boehmer, Claudia Bruguera Torres, Rachel Spivak, Max Chou, Kristie Geisler, Keith O'Conor, Sara E Williams, Lili Ding, Christopher D King, Vanessa A Olbrecht

**Affiliations:** 1 Cincinnati Children's Hospital Medical Center, 3333 Burnet Ave, Cincinnati, OH, 45229, United States, 1 5136364408; 2 The Ohio State University Wexner Medical Center, Columbus, OH, United States; 3 Department of Pediatrics, Vanderbilt University, Nashville, TN, United States; 4 Department of Surgery, Wayne State University, Detroit, MI, United States; 5 Department of Anesthesiology, University of Cincinnati Medical Center, Cincinnati, OH, United States; 6 Department of Anesthesiology, Penn State Milton S. Hershey Medical Center, Hershey, PA, United States; 7 Department of Anesthesiology, Perioperative and Pain Medicine, Stanford University, Palo Alto, CA, United States; 8 Department of Anesthesiology, Nemours Children’s Hospital, Wilmington, DE, United States; 9 Sidney Kimmel Medical College, Thomas Jefferson University Hospital, Philadelphia, PA, United States

**Keywords:** analgesia, anxiety, distraction-based virtual reality, pediatric anesthesia, prospective studies, child, human, virtual reality, pediatric pain medicine, acute postoperative pain

## Abstract

**Background:**

Virtual reality (VR) is a novel technology with implications for pain and sensory processing. VR may serve as a novel, scalable method to deliver clinically validated therapy for pain management as an alternative or adjunct to opioids for acute pain. Given that psychological factors and pain perception are both components of postoperative pain, it may also be beneficial to incorporate modalities that decrease anxiety, such as active relaxation and guided meditation with VR. Unfortunately, these therapies are not widely available due to multiple barriers. VR has the potential to deliver pain-reducing, psychologically based therapy to children, thereby enhancing multimodal analgesia and potentially decreasing opioid use. This study investigates the role of VR in reducing pain and anxiety after surgery. Given the substantial risks associated with opioid use, particularly in younger populations, alternative pain management strategies are crucial.

**Objective:**

The primary aim of this study was to evaluate the efficacy of VR as a nonpharmacological intervention for managing postoperative pain intensity, pain unpleasantness, anxiety, and opioid use in children and adolescents undergoing Nuss repair of pectus excavatum.

**Methods:**

A single-center, prospective, randomized, controlled trial was conducted at a tertiary care children’s hospital and research center. Ninety children and adolescents (8-18 y) undergoing the Nuss procedure were randomized to guided relaxation or mindfulness VR (n=30) and distraction-based gaming VR (n=30), combined to form the VR group (n=60), and a control group using a passive 360° video (n=30). Patients received a 10-minute session on postoperative days 1 and 2. Pain intensity, pain unpleasantness, and anxiety were evaluated before and 0-, 15-, and 30-minute post-session. In-hospital pain scores, anxiety scores, and opioid use were collected.

**Results:**

Children and adolescents who participated in VR reported a significantly greater decrease in pain intensity from baseline (0.41, SE 0.23) compared with those in the 360° video group at 30 minutes (*P*=.04) before multiplicity adjustment but not after multiplicity adjustment. There were no significant differences in pain scores or opioid use between the VR and control groups on postoperative day 1 or 2, nor were there changes in pain unpleasantness or anxiety at any time after the intervention.

**Conclusions:**

Daily, 10-minute VR sessions provided some trends toward transient analgesic and anxiolytic effects, albeit none that were statistically significant. VR did not significantly decrease overall pain scores or opioid usage, possibly due to the limited intervention duration and high standardized opioid use. Future studies should investigate extended and more frequent VR sessions and the integration of VR with other therapeutic modalities.

## Introduction

### Background

Multimodal pain management techniques for acute postoperative pain are commonly studied and utilized [1]; opioids continue to be the cornerstone of postoperative pain management. Opioid misuse continues to be a major public health issue in the United States, with children and adolescents particularly vulnerable, as many are initially exposed to opioids prescribed for pain management [2-4]. Furthermore, the risk of future opioid overdose significantly increases with the quantity of pills prescribed; adolescents receiving 30 or more pills have a 35% higher rate of overdose than those prescribed 18 or fewer pills [5]. The prescription of opioid analgesics is a well-documented pathway to misuse, opioid use disorder, and overdose [6].

The Nuss procedure, performed to repair pectus excavatum, is associated with severe postoperative pain [7]. Effective management of postoperative pain after this surgery is crucial, as alleviating pain can enhance patient satisfaction and reduce complication rates [8]. Effective pain management techniques and regimens vary across pediatric institutions and have begun including intercostal nerve cryoablation [8,9]. Opioid use during recovery from the Nuss procedure is common, with one study finding that patients used opioids for a median of 8 days with an IQR of 6‐10 days [10]. Given the absence of standardized postoperative pain management protocols and the high usage of opioids following the Nuss procedure, it is essential to explore nonpharmacologic pain control adjuncts for these patients.

Virtual reality (VR) technology provides an immersive, multisensory, and 3D environment that enables individuals to experience a modified reality, creating a sense of “presence” for each individual [11]. There is a clear need for alternative pain management methods, including nonpharmacologic techniques. VR has been shown to be effective in reducing perioperative and postoperative anxiety in pediatric patients. Studies show significant reductions in anxiety in pediatric patients immediately after distraction-based gaming virtual reality (VR-D) sessions, with some effects lasting for at least 15 minutes post-intervention [12,13]. Two approaches—VR-D and guided relaxation–based virtual reality (VR-GR)—are being researched for their effectiveness in reducing pain and anxiety following surgery.

VR-D immerses patients in engaging experiences that help divert attention from pain or anxiety, providing effective short-term relief. Integration of these techniques is challenging in the perioperative space, with limited providers and resources and high costs limiting its feasibility. VR can be used anywhere, anytime, with access to a headset.

Gate control theory suggests that distraction can be a valuable tool for pain management, as attentional load is fixed, and distraction toward a pleasant experience means less attention to pain [14,15]. It has been associated with immediate and short-term reductions in postoperative pain intensity and unpleasantness. VR-D techniques have been shown to decrease acute pain in children and adults [16-18]. Single sessions of VR-D have been shown to reduce postoperative pain for up to 30 minutes in some cases, regardless of baseline pain catastrophizing levels, suggesting broad applicability across pediatric populations experiencing postoperative pain [19]. The use of VR-D has also demonstrated pain reduction comparable to opioid use in burn injury patients during wound cleaning [20]. While VR-D is particularly effective for short-term pain management, additional strategies like guided relaxation may be needed for longer-lasting pain relief [21].

VR-GR seeks to provide more sustained pain relief by combining distraction with mind-body techniques, such as guided relaxation or mindfulness within the VR environment. Psychological factors—including calmness, fear, anxiety, and depression—affect the subjective experience of pain [22]. Resilience has been negatively associated with pain unpleasantness, potentially serving as a protective factor in patients with higher baseline anxiety [22,23]. Incorporating active relaxation and guided meditation techniques may significantly contribute to pain reduction. This combination of settling the mind to increase resilience and distraction from acute pain may play a significant role in acute pain reduction [21]. VR-GR may further improve anxiety reduction, especially in children with higher anxiety sensitivity [12,24]. Although VR-GR may offer additional benefits for sustained pain relief compared to distraction alone, its effects were also primarily transient [21]. Using VR to perform guided relaxation could expand the benefits of these nonpharmacological pain management techniques to more children, including those having surgery.

Overall, VR is a promising nonpharmacologic tool for managing postoperative pain and anxiety in children and adolescents. It can potentially enhance the perioperative experience, reduce reliance on pharmacological interventions, and increase patient and family satisfaction. However, randomized controlled trials are needed to establish standardized protocols and explore VR integration with other therapies, such as biofeedback, for more durable outcomes [13,21,25].

### Aim

In this prospective, randomized, controlled clinical trial, we compare the short-term efficacy of immersive VR in decreasing acute postoperative pain (primary outcome), anxiety, and opioid consumption following pectus excavatum repair. We hypothesize that the use of VR will be more effective at reducing pain, anxiety, and opioid use as compared to the control group in this patient population.

## Methods

The original protocol for this study has been published [26].

### Study Design and Setting

This single-center, randomized, unblinded clinical trial was conducted at Cincinnati Children’s Hospital Medical Center (CCHMC), a 670-bed tertiary care academic children’s hospital. The recruitment began on July 10, 2020, and the study was completed on July 30, 2023. The COVID-19 pandemic delayed study completion. We recruited children and adolescents undergoing Nuss repair of pectus excavatum to investigate the role of VR in the management of postoperative pain and anxiety.

### Ethical Considerations

This study complies with the Standard Protocol Items: Recommendations for Interventional Trials (SPIRIT) statement [27] and the Consolidated Standard of Reporting Trials (CONSORT) statement [28]. The CCHMC Institutional Review Board approved this study (IRB 2019‐1090) on November 26, 2019, and it was conducted per the rules and regulations for ethical research. This study was registered at ClinicalTrials.gov on April 3, 2020 (NCT04351776). Written informed parental consent and patient assent (children >11 years) were obtained from all participants before enrollment into this study. Patients received a small stipend for participation. All identifying patient information was kept private and confidential.

### Patients and Recruitment

#### Patients

This study recruited 90 patients (30 patients per group), ages 8 to 18 years, undergoing Nuss repair of pectus excavatum surgery. Informed parental consent and patient assent were obtained before enrollment into this study.

The inclusion criteria were as follows: patients were (1) between the ages of 8 and 18 years, (2) able to read, understand, and speak English, (3) presenting for Nuss repair of pectus excavatum, and (4) followed by the acute pain service following surgery.

The exclusion criteria were as follows: patients with (1) a history of developmental delay, uncontrolled psychiatric conditions, or neurological conditions, (2) a history of seizures, epilepsy, vertigo, or significant motion sickness/nausea/vomiting, or (3) any condition that would preclude the application of the VR headset, such as craniofacial abnormalities.

#### Recruitment

Approximately 150 Nuss repair surgeries are performed at CCHMC each year. Therefore, our recruitment target of 90 patients was well within achievable limits. During the study, patients who underwent Nuss repair were recruited continuously until we met the targeted enrollment. The operating room schedule and surgical patient list were reviewed for potentially eligible patients, who were approached for recruitment before surgery. If patients wished to participate, consent (and assent for patients >11 years of age) was obtained, and eligibility criteria were verified. We recruited about 2 patients per week. Recruiting stopped during the COVID-19 pandemic, when elective surgeries were not performed, delaying study completion.

### Randomization

Potential patients were identified using the operating room schedule and the pectus surgery list provided by the surgery team. Eligible participants were randomized (1:1:1) into three groups: active distraction-based guided relaxation virtual reality (VR-DGR, n=30) and active VR-D (n=30)—collectively the VR group (n=60)—and a control group—passive 360° video (360-V) without instructions, sound, guided relaxation, or active patient involvement (n=30).

### VR Technology

All participants used a Starlight Xperience VR all-in-one device and software developed specifically for hospital settings. This technology is a customized version of the Lenovo Mirage Solo with a Daydream VR headset. It is easy to disinfect to comply with hospital infection safety protocols. Importantly, an integrated headphone device provides audio content, and the patients use head movements and a handheld controller for interaction and navigation. It is commercially available (not Food and Drug Administration–regulated) and was supplied by the Starlight Children’s Foundation.

VR-DGR and 360-V participants used the Mindful Aurora application, developed by the Stanford University Childhood Anxiety Reduction through Innovation and Technology program, to deliver relaxation/mindfulness content, which presents a relaxing nature scene with prompts instructing patients to actively slow and pace their breathing in conjunction with the movement of objects in the VR environment. 360°-V participants experienced the same relaxing nature scene without guided relaxation prompts; the 360°-V group also did not receive any audio and thus did not experience an immersive environment.

**Figure 1.**
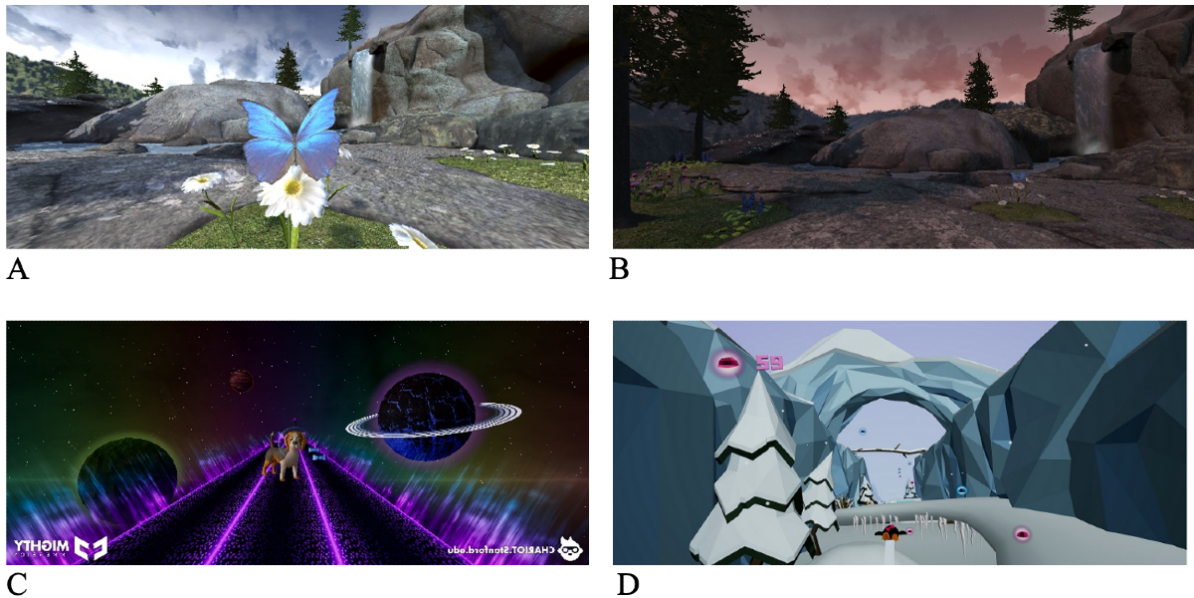
Scenes from the Mindful Aurora application used in distraction-based guided relaxation virtual reality (VR-DGR) and 360° video (360-V) (A and B), and scenes from Space Pups (C) and Pebbles the Penguin (D) used in distraction-based gaming virtual reality (VR-D).

VR-D participants had the option to choose and play one of the three games: Space Pups, in which the participant controls a puppy in space and collects treats to music; Pebbles the Penguin, in which the participant controls a penguin sliding down a mountain to collect pebbles; or Wonderglade*,* in which the participant can play five different mini-carnival games (Figure 1).  

### Procedure

Consent and assent were obtained before the visit. Patient characteristics, demographics, weight, and pain scores were collected preoperatively. All patients enrolled in this study received standard postoperative care via the CCHMC Pectus Surgery Pain Management Protocol, which standardizes all medications received by all pectus patients. This includes non-opioid pain medication such as pregabalin, acetaminophen, ketorolac, methocarbamol, and diazepam. All participants received the same non-opioid medications. Before the first session, patients completed the Childhood Anxiety Sensitivity Index to establish baseline anxiety levels and the Pain Catastrophizing Scale (PCS) for children. Patients were visited daily for one 10-minute session. Every effort was made to ensure the consistent timing of the visits for all patients. Sessions were completed beginning on postoperative day (POD) 1, then daily until the day of discharge, or until POD 3.

Patients were trained to use the technology before the first VR session. All participants received a device tutorial that taught them how to use the device and introduced them to the VR software. Patients received a script about VR-GR, VR-D, or 360-V, depending on the group to which they are assigned. During each session, patients completed a 10-minute session of either VR-GR, VR-D, or 360-V, per assigned group. Patients were asked to rate their pain intensity, pain unpleasantness, and anxiety via Numerical Rating Scale (NRS), before, immediately after, and 15 and 30 minutes after each session. Pain and anxiety scores and opioid use/day were recorded in REDCap (Research Electronic Data Capture).

### Data Collection

The primary outcome measure was pain intensity (NRS), measured before, immediately after, and 15 and 30 minutes following each session on POD1 and POD2. Secondary outcomes included opioid use on POD0, POD1, and POD2 and pain area under the curve (AUC) on POD1 and POD2, pain unpleasantness, and anxiety scores before and 0, 15, and 30 minutes after each session on POD1 and POD2 to establish change from baseline.

For each eligible participant, data were collected from their patient history/interview and the electronic medical record in a standardized case report form in the REDCap system. Inpatient opioid use was identified from the patient’s electronic medical record based on documentation in the medication administration record and transferred to REDCap. All opioid quantities were translated to morphine milligram equivalents (MME) and summed to determine total morphine equivalents per 24-hour period (midnight-to-midnight) during the patients’ inpatient stay. Measures used in the study are summarized in Table 1.

**Table 1.** Scales and questionnaires used in the study.

Scales and questionnaires	Definition
Pain intensity and pain unpleasantness	
Numerical Rating Scale (NRS) [29]	The NRS is the most common validated self-report measure of pain intensity and pain unpleasantness. It involves verbally asking for an estimate of pain intensity using numbers from 0 (no pain) to 10 (maximal pain). Pain was described as being like listening to music; pain intensity is the volume, and pain unpleasantness is how much the music is disliked [30]. It requires no equipment to administer or score.
Pain intensity across all postoperative days	Area under the time-pain score (NRS) curve using the trapezoidal rule (pain AUC^a^) measured pain intensity across all postoperative days 1 and 2.
Anxiety	
Pain Catastrophizing Scale for Children (PCS-C) [31]	A validated 13-item questionnaire (each rated on a 5-point scale, 0-4) designed to measure pain catastrophizing in children of age 8‐17 years. It is adapted from the adult version and assesses three key aspects of pain-related negative thinking: rumination, magnification, and helplessness.
Child Anxiety Sensitivity Index (CASI) [32]	A validated 18-item survey that measures perceived anxiety symptoms. Participants respond to each item on a 3-point scale (eg, “none,” “some,” and “a lot”). The total score is calculated by summing the responses, with higher scores indicating greater anxiety sensitivity. The total scores range from 18 to 54. CASI has been used in VR^b^ studies in adolescents of age 10‐21 years [33].
Numerical Rating Scale-Anxiety (NRS-A) [34]	A validated self-report numeric 0‐10 anxiety scale that is easy to administer to children. The NRS-A is easy to administer and can be used quickly to assess anxiety levels.
Opioid use	
NIH morphine milligram equivalents (MME) per day [35,36]	Standardizes a metric for quantifying and comparing doses of different opioids. However, MMEs serve as a common metric for comparing different opioids.

a AUC: area under the curve.

b VR: virtual reality.

### Statistical Analysis

#### Sample Size Calculation

Sample size was based on the feasibility of conducting this clinical study and unpublished preliminary data that assessed the impact of a single VR-D session on pain intensity in children and adolescents after surgery, with a goal of 80% power to detect differences in mean changes of 1 between VR and 360-V (given pilot data which showed average change in pain intensity of −1 [SD 1.2] and correlation of 0.88). Assuming similar results in the passive control group, a sample size of 30 per group will have 80% power to detect differences in mean changes of 1 between VR-GR and the two control groups. Significance (α) is .025 to control for 2 comparisons.

The VR-DGR and VR-D groups were combined into a single VR group for data analysis because both groups utilized active, distraction-based, immersive VR experiences. VR-DGR did not provide participants with feedback on their respiratory or heart rates. Consequently, it functioned as a distraction-based technique and did not significantly differ from the VR-D experience. Therefore, we combined the two groups, as both were fundamentally distraction-based.

A sample size of 60 for the treatment group and 30 for the control group will have 80% power to detect differences in mean changes of 1 between VR and control.

#### Data Analysis

All statistical analyses were performed using SAS 9.4 (SAS Institute). Patient demographics were described using mean (SD) or median (IQR) for continuous variables, depending on data distribution, and frequency (percentage) for categorical variables and compared between groups using *t* tests, Wilcoxon rank-sum tests, chi-square tests, or Fisher exact tests, as appropriate. Pain AUCs on POD1 and POD2 were calculated as the area under the time-pain score curve using the trapezoidal rule. MMEs, a metric for quantifying and comparing doses of different opioids, were derived for POD0, POD1, and POD2. Change from baseline on pain intensity, pain unpleasantness, and anxiety immediately after and 15 and 30 minutes following each session was calculated as the postinterval value minus the baseline (before session) value on POD 1 and POD2. Mixed effects models for repeated measures were used for pain AUC, MME, and change from baseline on pain intensity, pain unpleasantness, and anxiety outcomes. All mixed effects models included the intervention group and POD as fixed effects and the participant as a random effect. Models for the change from baseline outcomes also included baseline value, time (0-, 15-, and 30-min post-intervention), and group and time interaction as fixed effects. Missing data in the outcomes were examined for pattern and assumed missing at random and handled using full information maximum likelihood (FIML) for mixed effects models. Sidak adjustment for multiplicity was used for change from baseline in pain intensity between intervention groups at 3 time points (immediately after and 15 and 30 minutes following each session).

## Results

### Participants

**Figure 2.**
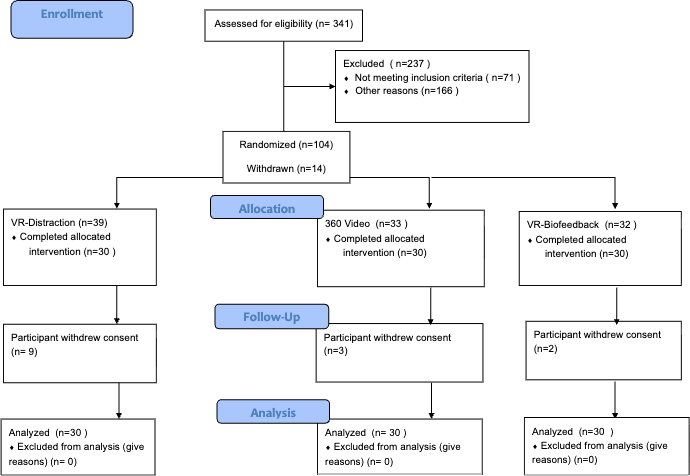
CONSORT (Consolidated Standards of Reporting Trials) 2010 flow diagram. VR: virtual reality.

Ninety patients were enrolled in the study (60 in VR and 30 in 360-V; Figure 2 ). The participants comprised 73 male and 17 female patients and had an American Society of Anesthesiologists (ASA) Physical Status Classification System score of 1‐3, with a mean age of 15.5 (SD 1.4) years. The 2 groups had no differences demographically except for a difference in PCS scores (VR: median 18, IQR 15‐23; VR-360: median 24, IQR 16‐28; *P*=.04). Patients were primarily male, adolescent, and Caucasian. This is consistent with the demographics of patients with pectus excavatum [37], and these are the patients most likely to undergo the Nuss procedure [38] (Table 2). All patients had at least 1 observation on all repeated-measured outcomes (pain AUC, MME, pain intensity, pain unpleasantness, and anxiety), and all available data were included in the mixed effects models for the outcomes.

**Table 2.** Patient characteristics.

Characteristic	VR^a^	360° video	Overall	*P* value (test)
Age (y), mean (SD)	15.6 (1.4)	15.1 (1.5)	15.5 (1.4)	.10
ASA^b^ physical status, n (%)				.34
I	3 (5)	4 (13.3)	7 (7.8)	
II	44 (73.3)	21 (70.0)	65 (72.2)	
III	13 (21.7)	5 (16.7)	18 (20.0)	
Race, n (%)				.55
Caucasian	57 (95)	30 (100)	87 (96.7)	
African American	0 (0)	0 (0)	0 (0)	
Asian	0 (0)	0 (0)	0 (0)	
Other	3 (5)	0 (0)	3 (3.3)	
Ethnicity, n (%)				.25
Hispanic	3 (5)	0 (0)	3 (3.3)	
Non-Hispanic	56 (93.3)	28 (93.3)	94 (93.3)	
Unknown	1 (1.7)	2 (6.7)	3 (3.3)	
Sex, n (%)				.70
Male	48 (80)	25 (83.3)	73 (81.1)	
Female	12 (20)	5 (16.7)	17 (18.9)	
Weight (kg), mean (SD)	58.9 (9.8)	56.3 (10)	58.1 (9.9)	.24
CASI^c^ score, mean (SD)	29.3 (5)	30.9 (4.8)	29.8 (5)	.14
Pain Catastrophizing Scale (PCS), median (IQR)	18 (15-23)	24 (16-28)	19 (15-26)	.04

a VR: virtual reality.

b ASA: American Society of Anesthesiologists.

c CASI: Child Anxiety Sensitivity Index.

### Changes From Baseline (VR vs Control)

#### Pain Intensity

**Figure 3.**
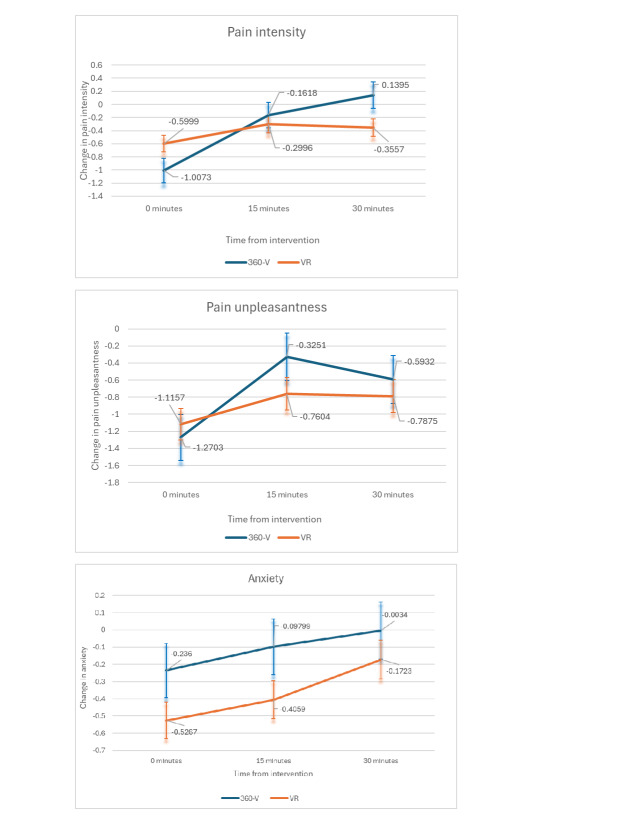
Changes in baseline in pain intensity, pain unpleasantness, and anxiety in time points following 360° video (360-V) and virtual reality (VR) in a mixed effect model with standard error bars.

Patients who participated in VR reported significantly decreased pain intensity from baseline (0.41 more decrease in pain intensity with SE 0.23) compared with those in the 360-V group at 30 minutes (*P*=.04) before multiplicity adjustment but not after multiplicity adjustment. There was no significant difference from baseline in reported pain intensity between VR vs 360-V immediately following the session (*P*=.08) or after 15 minutes (*P*=.56; Figure 3).

#### Pain Unpleasantness

There was no significant difference in the reported pain unpleasantness between patients who participated in VR versus 360-V immediately following the VR session (*P*=.64), after 15 minutes (*P*=.20), or after 30 minutes (*P*=.57; Figure 3).

#### Anxiety

There were no significant differences in reported anxiety from baseline between patients who participated in the VR versus 360-V immediately following the session (*P*=.13), after 15 minutes (*P*=.12), or after 30 minutes (*P*=.40; Figure 3).

#### Inpatient Pain and Opioid Use

There were no significant differences in mean AUC pain scores between VR and 360-V (*P*=.60). There were also no significant differences in inpatient opioid use (MME/kg/day) between VR and 360-V (*P*=.26; Table 3).

**Table 3.** Inpatient pain and opioid use.

Characteristic	VR^a^, mean (SD)	360-V^b^, mean (SD)	*P* value^c^
Inpatient pain (AUC^d^)			.60
POD^e^1	110.0 (34.3)	107.9 (31.8)	
POD2	95.9 (32.5)	90.6 (36.0)	
Inpatient opioid use (MME^f^/kg/day)			.26
POD0	0.18 (0.29)	0.18 (0.24)	
POD1	0.58 (0.22)	0.64 (0.28)	
POD2	0.50 (0.17)	0.51 (0.20)	

a VR: virtual reality.

b 360-V: 360° video.

c *P* value from mixed effects models.

d AUC: area under the curve.

e POD: postoperative day.

f MME: morphine milligram equivalents.

## Discussion

### Principal Results

In our study, we found that active, immersive VR experiences had some trends to transient effects on both acute pain and anxiety compared to a nonimmersive 360-V control; however, these effects did not meet statistical significance. Patients who participated in VR reported a significantly greater decrease in pain intensity from baseline (0.41 with SE 0.23) compared with those in the 360-V group at 30 minutes (*P*=.04) before multiplicity adjustment but not after multiplicity adjustment. The trends in reduction in pain and anxiety were small; these trends did not achieve clinical significance either. Current literature indicates that a reduction of at least 2 points on the NRS for pain intensity or a 30% reduction in pain is considered clinically significant [39]. We did not see effects on overall AUC pain scores or opioid use. In this research, the VR-GR experience was likely distraction-based, as we could not document or assess feedback on patients’ ability to perform the guided relaxation techniques correctly. In spite of not reaching statistical significance, these trends are not an absence of evidence of the effectiveness of VR to reduce postoperative pain and anxiety. We had relatively similar treatment conditions in small samples with attrition. The trends point us in the direction of future work.

Demographically, our two groups showed no significant differences except for a difference in PCS scores (VR: median 18, IQR 15‐23; VR-360: median 24, IQR 16‐28; *P*=.04). However, this result, while statistically significant, may not be clinically significant, as only PCS scores above 30 are clinically relevant, and neither group’s median score exceeded 30 [40].

Although we noted some trends toward reduction in acute pain and anxiety from immersive VR following Nuss repair of pectus excavatum, these effects did not result in a significant change in AUC pain scores or inpatient opioid usage between the two groups. Several factors may account for the lack of significance. At our institution, the standard postoperative pain management protocol for following pectus surgery involves the scheduled administration of opioids, meaning all patients receive a standardized, weight-based dosage of opioids during their hospital stay, regardless of their actual pain level, with variations only in as-needed doses [10]. Consequently, opioid consumption may not accurately represent the patients’ pain levels and opioid requirements. Home medication use might better reflect patients’ pain and opioid needs. This study limited VR experiences to hospitalized patients. Extending its use past hospital discharge may have yielded different results. Future studies should investigate the integration of VR therapy into postoperative pain management both during and after hospitalization.

### Limitations

Although this study was a prospective, randomized clinical trial, which can provide the best clinical evidence and support for VR, it has several limitations due to both study design and factors outside the control of the research team. Our patient population was somewhat homogeneous, as most individuals undergoing pectus excavatum repair are adolescent white males [38]. Therefore, our findings may not be generalizable to a broader population. Additionally, our control group may have been too similar to our intervention group. While the 360-V group did not receive audio instructions for guided relaxation, they still used a VR headset and experienced some level of distraction and immersion. Hence, the difference between the two groups may not have been substantial enough to detect a meaningful difference. This lack of significant differences in the treatment groups may account for the lack of statistical differences between the two groups when comparing pain and anxiety. Future research will use nonimmersive, non-headset control and retrospectively compare historical data to better assess the impact of VR on these outcomes.

Reporting bias may have also played a role, as study participants may have felt inclined to report decreased pain and/or anxiety after treatment due to their perception of receiving an intervention, regardless of actual changes. The self-reported 1 to 10 rating scale is limited and has been shown to have a moderate correlation with clinical indicators of pain; thus, we cannot rely exclusively on this measure for pain evaluation [41]. Future studies should consider additional outcome measures.

The limited use of the intervention (10 min per d) makes it unlikely to produce meaningful changes in the severe postoperative pain often experienced by these patients. This suggests a need for further investigation of VR for pain relief, potentially incorporating VR interventions more systematically throughout the perioperative period. This could include preoperative VR exposure, repeated interventions multiple times per day, for additional consecutive days following surgery. We accounted for our attrition in our statistical analysis. However, the attrition with small groups to start likely was one reason for our inability to find statistically significant differences in results between the two groups.

### Comparison With Prior Work

Controlling pain after surgery is important; uncontrolled postoperative pain can lead to increased morbidity, decreased function, prolonged recovery, and higher costs [42]. Severe acute postoperative pain can also lead to chronic postoperative pain, with rates of chronic postsurgical pain reported to be about 20% in pediatric populations [43]. Furthermore, opioid use after surgery also has risks, including persistent opioid use postoperatively; one study found a rate of 4.8% of persistent opioid use in postoperative adolescents as compared to a 0.1% rate of persistent opioid use in their nonsurgical matched cohorts [44].

Our study’s results align with prior research suggesting that VR-D may play a role in transiently reducing acute pain and that relaxation via guided imagery can promote reductions in both pain and anxiety [20,45]. Research consistently shows that VR is effective in lowering procedural pain, anxiety, and fear in pediatric patients, particularly during needle-related and other painful interventions [46-55]. A few studies have also demonstrated the feasibility of using VR to alleviate acute postoperative pain [21,56]. In adults, a meta-analysis found that patients receiving perioperative VR had lower pain scores than those receiving usual care (mean deviation −0.64, 95% CI −1.05 to −0.22; *P*<.02). Additionally, patients receiving VR postoperatively experienced a significant reduction in pain scores (mean deviation −0.50, 95% CI −0.76 to −0.24; *P*=.002) [57]. One pediatric study indicated that a single preoperative VR experience reduced the need for rescue analgesics in the recovery unit for painful procedures [58].

Relaxation-guided imagery has been shown to reduce both pain and anxiety in children undergoing minor surgery [59]. VR-GR has also demonstrated effectiveness in reducing pain and anxiety in children during medical procedures. These effects were immediate but transient, with some studies reporting reductions lasting up to 30 minutes after a session [21,60,61]. Additionally, a small (n=51) single-center, prospective study evaluated a single VR-GR session for acute postoperative pain and anxiety in children and adolescents. This study showed similar transient reductions in pain intensity and anxiety [21].

Using guided relaxation, our study aimed to harness the benefits of using mind-body techniques to manage postoperative pain. However, there are several potential reasons for the lack of clinically significant lasting effects on acute pain and anxiety in this population or opioid consumption. First, our study did not include a non-headset control group. Previous studies assessing the effects of VR on opioid use compared VR experiences to standard care without VR [55,62,63]. In our study, every patient utilized a VR headset and had some level of an immersive experience, even with 360-V (a nonimmersive option). As a result, all patients, including the control group, likely experienced some distraction.

Moreover, the effectiveness of the VR sessions may have been limited by the short duration of each session and the low number of sessions. Ten minutes per day may be an inadequate time to achieve lasting pain relief of severe acute pain, especially as compared to other therapeutic and/or pharmacologic interventions that are administered more frequently. Future studies should investigate the optimal timing and length of intervention for maximal benefit of VR.

While our results indicated that active, immersive VR experiences had some trends toward effects on both acute pain and anxiety compared to the nonimmersive 360-V control, these effects were not clinically significant. The study may not have produced clear positive results due to experimental factors such as the similarity of the VR and control treatments, the short duration of treatments, and the protocol-driven high use of opioids that were not adequately considered in the study design. Nevertheless, the findings provide a valuable framework for designing future VR studies. We can learn from our null results to design future VR studies with a control treatment that does not use a VR headset and use a population that only receives opioids on an as-needed basis. Longer treatment duration and less subject attrition could also lead to more significant results.

### Conclusions

This study found that daily, 10-minute VR sessions had trends toward transiently reducing pain and anxiety compared to a 360-V experience in participants following Nuss repair of pectus excavatum. These results were not clinically significant. Due to the limited duration of the intervention and the standardized, scheduled, high utilization of opioids in this population, VR was not sufficient in significantly decreasing opioid use and overall AUC pain scores. Despite these conclusions, exploring guided relaxation VR as an adjunct to, rather than a replacement for, postoperative pharmacologic analgesics may prove valuable. Increasing the length and frequency of VR experiences per day, along with a policy of not automatically administering opioids unless requested, may help decrease opioid usage and AUC pain scores. A systematic integration of VR into perioperative care is likely necessary to impact the pain trajectory and opioid usage in postoperative patients. Furthermore, improving the VR experience to incorporate true guided relaxation would likely enhance effectiveness compared to a purely distraction-based approach. Future studies are needed to further explore the use of this therapy in postoperative pain management.

## Supplementary material

10.2196/80902 Checklist 1 CONSORT-eHEALTH checklist (V 1.6.1).

## Abbreviations

360-V: 360° video
AUC: area under the curve
CCHMC: Cincinnati Children’s Hospital Medical Center
CONSORT: Consolidated Standard of Reporting Trials
MME: morphine milligram equivalents
NRS: numerical rating scale
PCS: Pain Catastrophizing Scale
POD: postoperative day
REDCap: Research Electronic Data Capture
SPIRIT: Standard Protocol Items: Recommendations for Interventional Trials
VR: virtual reality
VR-D: distraction-based gaming virtual reality
VR-DGR: distraction-based guided relaxation virtual reality
VR-GR: guided relaxation–based virtual reality
